# Distribution and postnatal development of chondroitin sulfate proteoglycans in the perineuronal nets of cholinergic motoneurons innervating extraocular muscles

**DOI:** 10.1038/s41598-022-25692-3

**Published:** 2022-12-14

**Authors:** Adrienn Ritok, Peter Kiss, Anas Zaher, Ervin Wolf, Laszlo Ducza, Timea Bacskai, Clara Matesz, Botond Gaal

**Affiliations:** 1 Department of Anatomy, Histology and Embryology, Faculty of Medicine, University of Debrecen, 4032 Debrecen, Hungary; 2 Division of Oral Anatomy, Faculty of Dentistry, University of Debrecen, 4032 Debrecen, Hungary; 3 Department of Obstetrics and Gynecology, Kenezy Gyula Campus of the University of Debrecen, Faculty of Medicine, 4032 Debrecen, Hungary; 4 New York Presbyterian Brooklyn Methodist Hospital, 506 6th St, Brooklyn, NY USA

**Keywords:** Neuroscience, Molecular neuroscience, Motor control, Neural circuits, Oculomotor system

## Abstract

Fine control of extraocular muscle fibers derives from two subpopulations of cholinergic motoneurons in the oculomotor-, trochlear- and abducens nuclei. Singly- (SIF) and multiply innervated muscle fibers (MIF) are supplied by the SIF- and MIF motoneurons, respectively, representing different physiological properties and afferentation. SIF motoneurons, as seen in earlier studies, are coated with chondroitin sulfate proteoglycan rich perineuronal nets (PNN), whereas MIF motoneurons lack those. Fine distribution of individual lecticans in the composition of PNNs and adjacent neuropil, as well as the pace of their postnatal accumulation is, however, still unknown. Therefore, the present study aims, by using double immunofluorescent identification and subsequent morphometry, to describe local deposition of lecticans in the perineuronal nets and neuropil of the three eye movement nuclei. In each nucleus PNNs were consequently positive only with WFA and aggrecan reactions, suggesting the dominating role of aggrecan is PNN establishment. Brevican, neurocan and versican however, did not accumulate at all in PNNs but were evenly and moderately present throughout the neuropils. The proportion of PNN bearing motoneurons appeared 76% in oculomotor-, 72.2% in trochlear- and 78.3% in the abducens nucleus. We also identified two morphological subsets of PNNs, the focal and diffuse nets of SIF motoneurons. The process of CSPG accumulation begins just after birth, although considerable PNNs occur at week 1 age around less than half of the motoneurons, which ratio doubles until 2-month age. These findings may be related to the postnatal establishment of the oculokinetic network, performing different repertoires of voluntary eye movements in functionally afoveolate and foveolate animals.

## Introduction

Extracellular spaces in the central nervous system (CNS) contain numerous macromolecules of the extracellular matrix (ECM). In the adult brain, ECM can be present in various forms: (i) across the neuropil diffuse matrix loosely fills narrow intercellular gaps; (ii) ECM condenses around the presynaptic axons forming the axonal coat; (iii) and may also accumulate in the nodes of Ranvier as nodal ECM. (iv) Around neuronal somata and processes dense ECM coats build up forming the perineuronal nets (PNN), dominantly built by lecticans (aggrecan, brevican, neurocan, versican) of the chondroitin sulfate proteoglycans (CSPG)^1–3^. Core proteins of lecticans anchor to a hyaluronan backbone at their N-terminus and are crosslinked by tenascins at the C-terminus. The molecular composition of PNNs shows unique regional distribution, correlating with the morphology, neurochemical properties, connections and functions of local neurons^4–12^. ECM—including the diffuse form and PNNs—is a dynamic component of the nervous tissue, playing an essential role in material trafficking, transmembrane signaling, tissue (re)modelling, network development, peri- and transsynaptic structural support. Training- and lesion experiments suggested the altered activity of neuronal circuits that initiate modifications within the PNN’s molecular assembly which enables a higher plasticity and perhaps functional repair^11,13–18^.

Prior hodological works on the oculomotor system have clarified that extraocular muscles are dually innervated by two anatomically and neurochemically segregated groups of motoneurons, encompassed by the oculomotor-, trochlear- and abducens nuclei, localized in the mesencephalon and pons^19–21^. Extraocular muscle fibers are classified according to their functional- and histochemical properties: (i) Singly innervated extraocular muscles fibers (SIF) are controlled by SIF motoneurons that occupy the central part of each eye movement nucleus. These cholinergic neurons provide the major portion of eye moving motoneurons. SIF motoneurons have long been characterized by their parvalbumin- and non-phosphorylated neurofilament positive somata, which were coated with perineuronal nets^22–24^. Muscle fibers innervated by SIF motoneurons correspond to the ‘all-or-nothing’ twitch muscle fibers of mammalian skeletal muscles^25^. (ii) The multiply innervated extraocular muscle fibers (MIF) are supplied by multiple terminals of MIF motoneurons, which provide only the minority of eye moving motoneurons and are localized in the periphery of each eye movement nucleus. MIF motoneurons are also acetyl cholinergic, but opposite to SIFs, MIF motoneurons are not enveloped by PNNs, nor do they express non-phosphorylated neurofilaments. Parvalbumin immunoreaction is either negative or weak in a fewer number of MIF neurons^20,22,23^. MIF motoneurons control the global non-twitch- and the combined orbital layer of extraocular muscle fibers by local tonic tension^26–31^.

The segregating neurochemical characteristics of SIF- and MIF motoneurons remained conservative throughout ontogeny, found uniform in monkeys^23^, rats^20^, and mice^22^. Of note, Eberhorn et al.^20^ suggests that the two motoneuron populations strongly segregate spatially in primates but overlap in rodents, which has also won evidence by the experiments of Bohlen et al.^22^ in mice.

In previous studies, the PNNs of the extraocular motoneurons were recognized either with the general markers of CSPGs, or by labeling their aggrecan component^22,23^ however no data is yet available on the distribution of other lecticans in their PNNs, nor is there quantitative data on the expression of lecticans in the oculomotor-, trochlear- and abducens nuclei. Knowledge on the lectican composition of PNNs surrounding the eye moving motoneurons is essential, as the diverse roles of individual lectican molecules were reported in various parts of the CNS during plastic adaptations or postlesional repair^17,32,33^.

The postnatal (PN) establishment of CSPG-based PNNs has also not been described earlier in the eye movement nuclei, which could contribute to understanding those critical periods of CNS development when the plasticity of a neural network dramatically reduces, terminating the wiring of the oculokinetic system and its synaptogenesis.

Based on immunohistochemical quantifications, this study aims to describe (i) the distribution of lectican molecules in the PNNs of SIF motoneurons; (ii) the differences in the expression of lecticans by statistical analysis of PNN optical densities; (iii) the local specificity of lectican accumulations in PNNs of motoneurons and neuropil in the oculomotor-, trochlear- and abducens nuclei of mice. (iv) The sequence of CSPG accumulation to forming PNNs has also been screened and statistically compared between postnatal day 1-to-2-month ages.

## Results

### In adult animals, only *WFA* and *aggrecan* reactions outlined perineuronal nets around the motoneurons of eye movements

**Figure 1:**
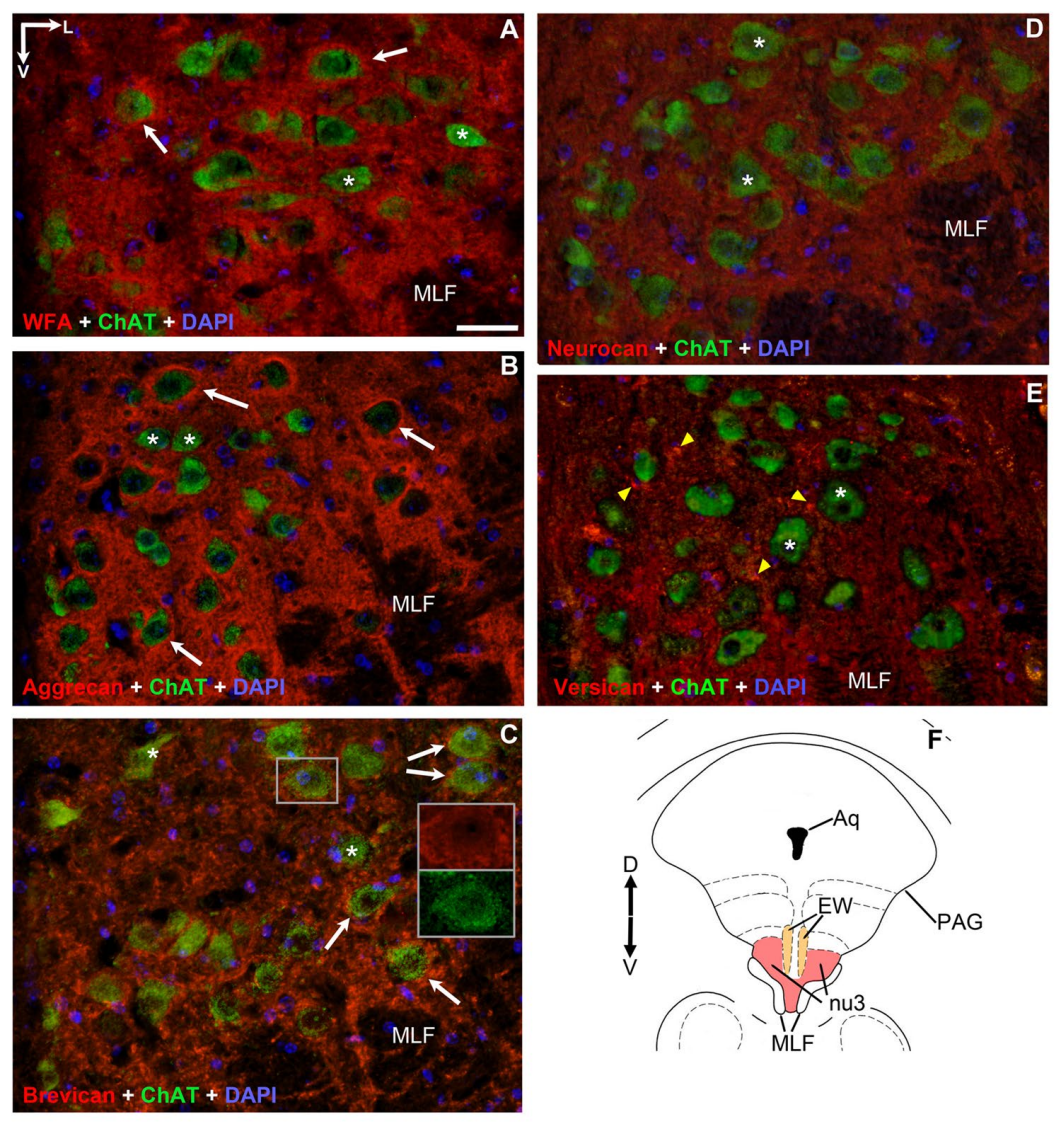
Pattern of chondroitin sulfate proteoglycans around acetyl-cholinergic motoneurons (green) in the *oculomotor nucleus*. The nuclei were detected with DAPI. PNNs (red) were immuno-positive with WFA and aggrecan labeling (white arrows) (**A**,**B**). PNNs were absent around only the minority of cholinergic motoneurons labelled with WFA and aggrecan (**A**,**B**), and were entirely absent with brevican, neurocan and versican immuno-staining (asterisks) (**C**–**E**). Nevertheless, rod-like brevican accumulations gathered in the proximity of some motoneurons (**C**), although PNNs did not assemble. Somata in (**C**) appear in yellow tone due to the co-labelling of the cytoplasmic brevican and acetylcholine. In (**E**) dot-like accumulations of versican characterized the neuropil (yellow arrowheads). (**F**) Position of oculomotor- (nu3) and Edinger-Westphal (EW) nuclei in the mesencephalon (modified after Paxinos and Franklin^35^) Aq: cerebral aqueduct. MLF: medial longitudinal fasciculus. PAG: periaqueductal gray matter. D: dorsal. V: ventral. L: lateral. Scalebar: 10 µm.

**Figure 2:**
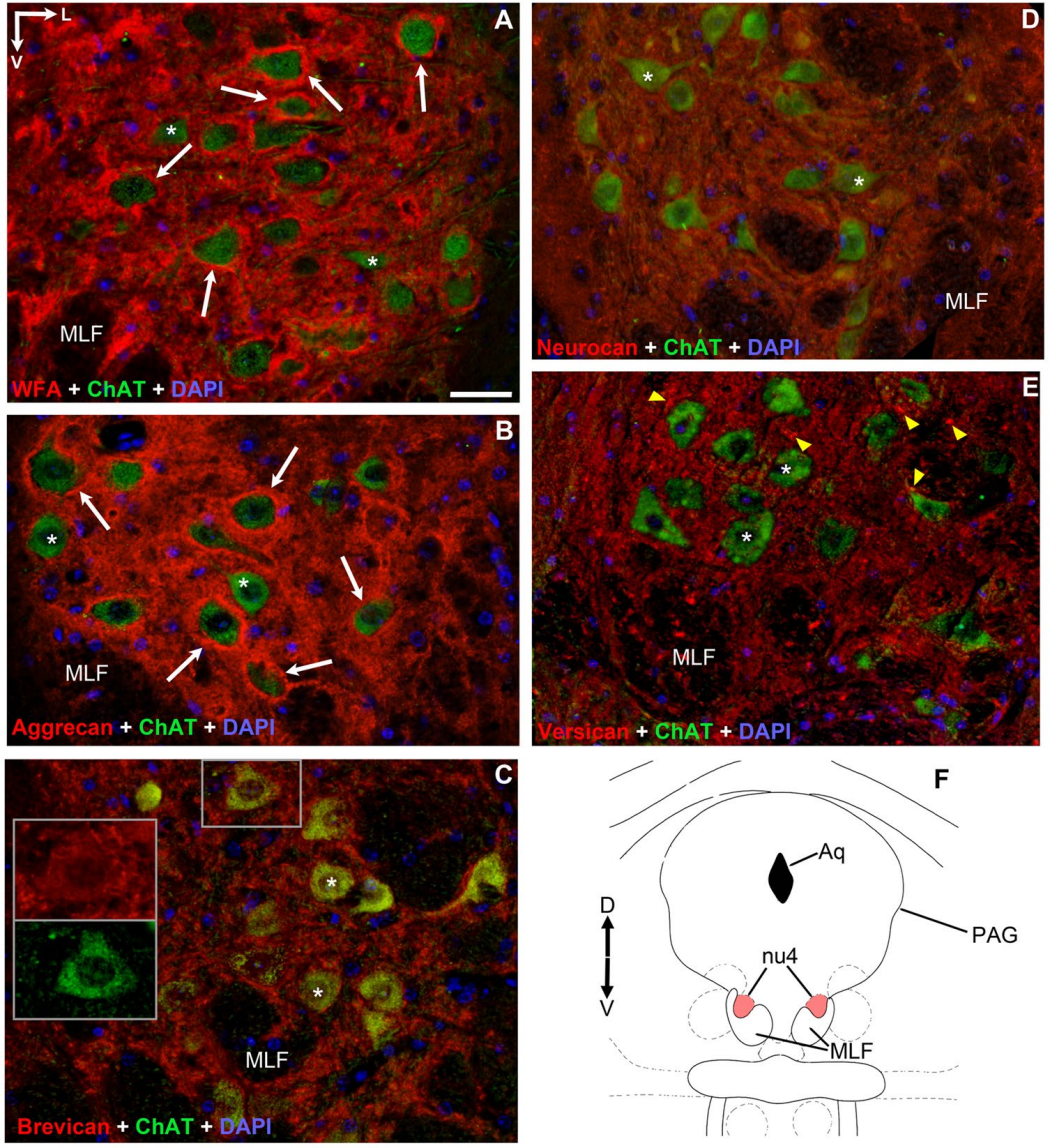
Pattern of chondroitin sulfate proteoglycans around acetyl-cholinergic motoneurons in the *trochlear nucleus*. PNNs (red) were only immuno-positive with WFA and aggrecan labeling (white arrows) (**A**,**B**). PNNs were absent around only the minority of cholinergic motoneurons labelled with WFA and aggrecan (**A**,**B**), and were entirely absent with brevican, neurocan and versican immuno-staining (asterisks) (**C**–**E**). Somata in (**C**) appear in yellow tone resulted by the co-labelling of cytoplasmic brevican and acetylcholine. In (**E**) dot-like accumulations of versican characterized the neuropil (yellow arrowheads). (**F**) Position of trochlear nucleus (nu4) in the mesencephalon (modified after Paxinos and Franklin^35^) Aq: cerebral aqueduct. MLF: medial longitudinal fasciculus. PAG: periaqueductal gray matter. D: dorsal. V: ventral. L: lateral. Scalebar: 10 µm.

**Figure 3:**
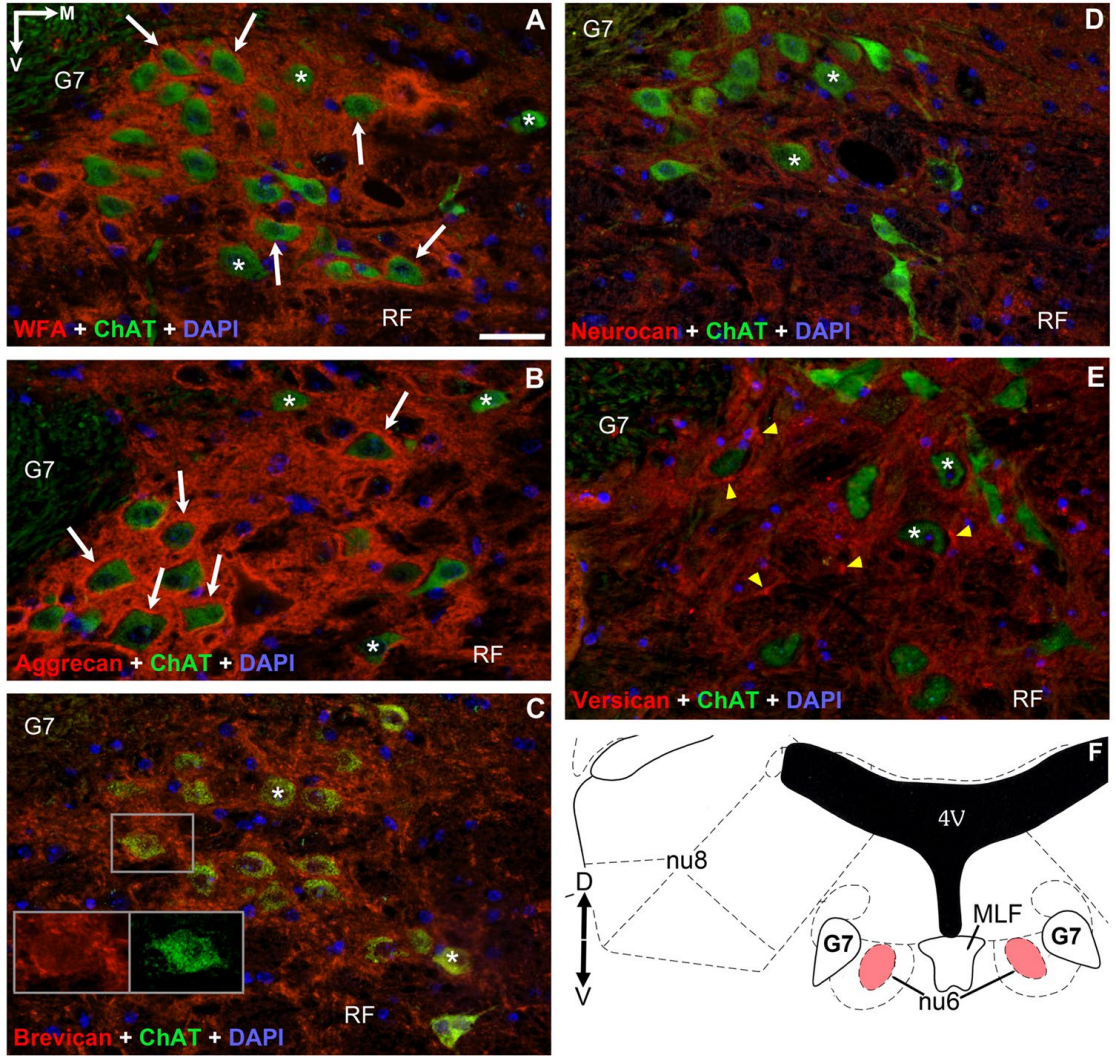
Pattern of chondroitin sulfate proteoglycans around acetyl-cholinergic motoneurons in the *abducens nucleus*. PNNs were only immuno-positive with WFA and aggrecan labeling (white arrows) (**A**,**B**). PNNs were absent around only the minority of cholinergic motoneurons labelled with WFA and aggrecan (**A**,**B**), and were entirely absent with brevican, neurocan and versican immuno-staining (asterisks) (**C**–**E**). Somata in (**C**) appear in yellow tone resulted by the co-labelling of cytoplasmic brevican and acetylcholine. In (**E**) dot-like accumulations of versican characterized the neuropil (yellow arrowheads). (**F**) Position of abducens nucleus (nu6) in the pons (modified after Paxinos & Franklin, 2001) 4V: 4th ventricle. MLF: medial longitudinal fasciculus. G7: facial genu. RF: reticular formation. nu8: vestibular nuclei. D: dorsal. V: ventral. M: medial. Scalebar: 10 µm.

**Figure 4:**
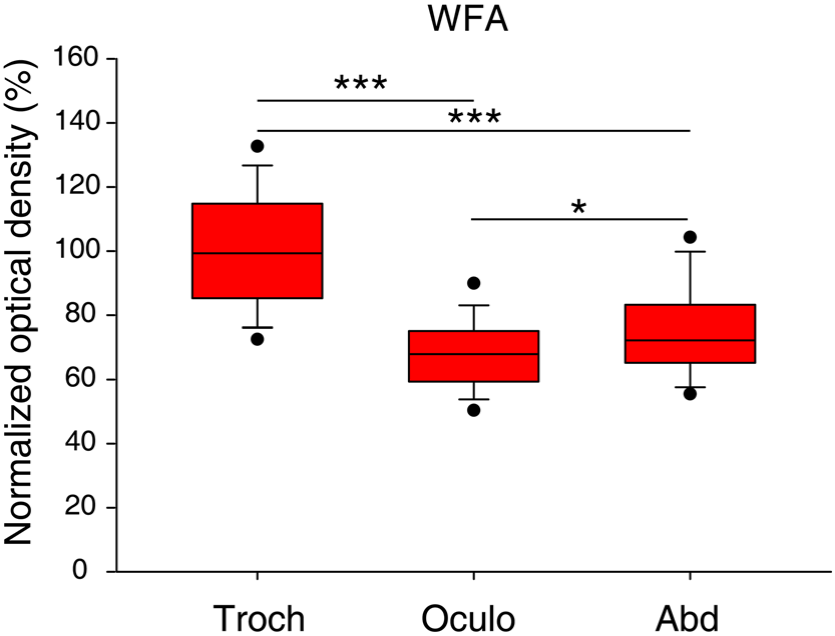
Optical densities of WFA reactions in the trochlear-, oculomotor- and abducens nuclei. Optical densities were normalized by the mean optical density of PNNs in the trochlear nucleus and expressed as percentages of this mean. Box plots represent the 10th, 25th, 50th 75th and 90th percentile values, lower and upper closed circles mark the 5th and 95th percentiles. There is a statistically significant difference in optical densities among the three nuclei (Kruskal–Wallis ANOVA on Ranks test, p < 0.001). Pairwise comparisons of optical densities revealed statistically significant difference between each pair of nuclei (Dunn’s post hoc test, ***p < 0.001, *p = 0.03).

**Figure 5:**
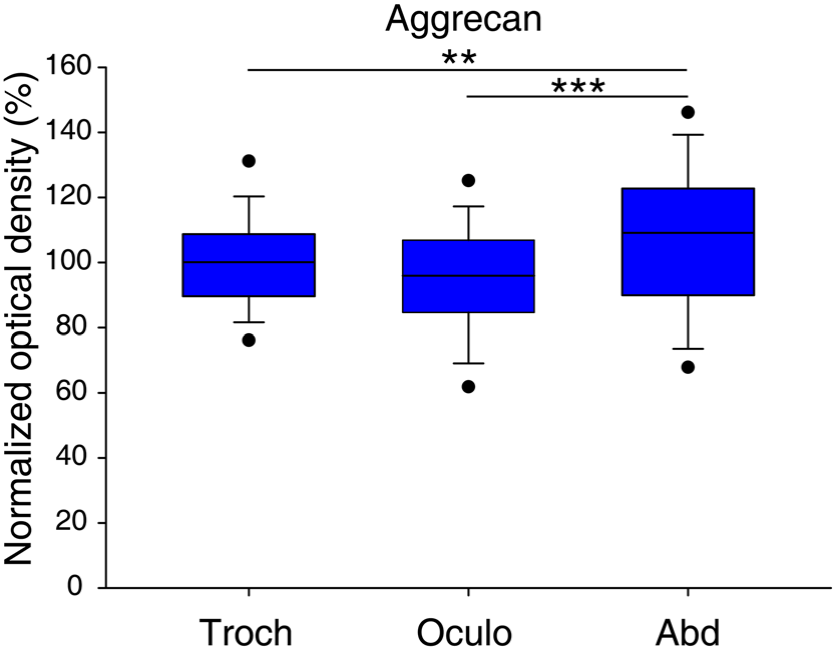
Optical densities of aggrecan reactions in the trochlear-, oculomotor- and abducens nuclei. Optical densities were normalized by the mean optical density of PNNs in the trochlear nucleus and expressed as percentages of this mean. Box plots represent the 10th, 25th, 50th 75th and 90th percentile values, lower and upper closed circles mark the 5th and 95th percentiles. There is a statistically significant difference in optical densities among the three nuclei (Kruskal–Wallis ANOVA on Ranks test, p < 0.001). Pairwise comparisons of optical densities showed statistically significant difference between two pairs of nuclei (Dunn’s post hoc test, oculomotorius vs. abducens ***p < 0.001 and trochlear vs. abducens **p = 0.007). No statistically significant difference was found between optical densities of trochlear- and oculomotor nuclei (Dunn’s post hoc test, p = 0.146).

Motoneurons of the oculomotor- and trochlear nuclei are situated in the ventral-most part of periaqueductal gray matter of the midbrain, whereas the abducens nucleus is found in the caudal pons, medially contacting to the facial genu (Figs. 1F, 2F, 3F)^34,35^. The general marker of CSPGs, as well as PNNs^1^, the *WFA*, showed very intense labeling both in the neuropil and perineuronal nets of the oculomotor, trochlear and abducens nuclei (Figs. 1A, 2A, 3A). PNNs were only recognizable around a distinct population of cholinergic neurons in each nucleus. The brightness intensity of PNNs was variable within a given nucleus and between the three nuclei, as revealed by quantitative analysis of the optical density values. The average brightness intensity of WFA labelled PNNs was the highest in the trochlear nucleus (100 ± 1.7%, n = 125), followed by the abducens- (74.9 ± 1.6%, n = 77) and lowest in the oculomotor nucleus (68.2 ± 1.1%, n = 114) (Fig. 4). Differences in the median values of WFA optical densities among the nuclei was greater than would be expected by chance (Kruskal–Wallis One Way Analysis of Variance on Ranks, p ≤ 0.001). Similarly, the *aggrecan* reaction was very intense in both the perineuronal nets and neuropil of each eye moving motor nuclei, in fact demarcation of the lattice-like pericellular area from the surrounding neuropil was more pronounced by using the *aggrecan* antibody (Figs. 1B, 2B, 3B). The tendency of mean brightness intensity was similar to the WFA reaction in the sense that the lowest optical density was measured in PNNs of the oculomotor nucleus (95.3 ± 1.2%, n = 238). Contrary to WFA, aggrecan reaction showed the highest optical density in PNNs of the abducens nucleus (107.7 ± 2.2%, n = 106), followed by the trochlear neurons (100 ± 1.2%, n = 174) (Fig. 5). The differences appeared statistically significant among the median values of the aggrecan reactions’ optical densities (Kruskal–Wallis One Way Analysis of Variance on Ranks, p ≤ 0.001).

### *Anti-brevican*, -*neurocan* and -*versican* reactions did not show perineuronal nets around motoneurons of eye movements in adult mice

Opposite to WFA and aggrecan, none of the reactions against brevican, neurocan or versican outlined PNNs around the motoneurons in any of the three nuclei, though each reaction exhibited its own characteristic pattern, as previously experienced in other regions of the CNS. Thus, *brevican* immuno-staining was moderate in each nucleus, distributed evenly in the neuropil. Its marble-like pattern consisted of strongly immunoreactive patches of 1–2 µm size, interrupted by low or unstained areas of the same dimensions. Patches gathered around somata, though continuous rings of PNN have not been identified (Figs. 1C, 2C, 3C). The density of brevican accumulates was highest in the oculomotor nucleus, followed by abducens nucleus and least seen in trochlear nucleus. Each cholinergic soma consequently exhibits yellow fluorescent staining, which phenomenon suggests the co-appearance of intracellular brevican and ChAT fluorescent signals (Figs. 1C, 2C, 3C inserts).

Moderately weak presence of *neurocan* was recognized only in the neuropil (Figs. 1D, 2D, 3D) of all three nuclei, distributed diffusely and homogenously, without demarcating any PNNs. Unlike in the case of brevican and versican labeling, heavily immune-positive accumulations of neurocan were characteristically absent both in the neuropil and perisomatic regions in each eye movement nucleus.

In agreement with earlier findings of Rácz et al.^2^, *versican* typically occurs as a mosaic of heavily- or lightly stained dots in the neuropil (Figs. 1E, 2E, 3E). The number and intensity of heavily immuno-positive spots are highest in the trochlear nucleus, and they seem lowest in the abducens nucleus. Although many of the dots gather in the proximity of cell bodies, none of those assemble into solid perineuronal nets. Of note, numerous dots were seen also in the medial longitudinal fasciculus (MLF) and internal facial genu (Figs. 2E, 3E).

### PNN-bearing- and non-bearing cholinergic motoneurons show similar proportions but different topography among the extraocular motor nuclei in adult mice

With WFA, the proportion of PNN bearing to all motoneurons appeared 76% in the oculomotor-, 72.2% in trochlear- and 78.3% in the abducens nucleus (Fig. 9). These proportions of motoneurons with and without PNNs were not proved to be significantly different among the three nuclei χ^2^(2, n = 631) = 1.86, p = 0.394).

**Figure 6:**
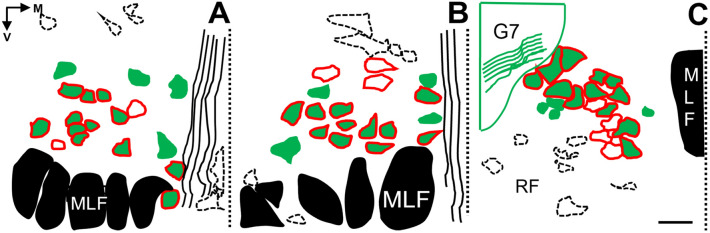
Topographical reconstruction of PNN-bearing- and non-PNN-bearing cholinergic motoneurons in the oculomotor- (**A**), trochlear- (**B**), and abducens nuclei (**C**). In (**A**) and (**B**), PNN bearing motoneurons are evenly distributed in the nuclei, whereas PNN non-bearing motoneurons concentrate in their medial aspects, or are loosely scattered centrally, dorso-medially and dorso-laterally. In **C**, PNN coated cholinergic neurons are densely fitted adjacent to each other, whereas non-PNN coated motoneurons are irregularly scattered in the nucleus or in its periphery. MLF: medial longitudinal fasciculus. V: ventral. M: medial. G7: facial genu. RF: reticular formation. Dotted line: midline. Frames of interrupted lines represent CSPG densities of non-cholinergic structures in periaqueductal gray matter (**A**,**B**) or pontine RF (**C**). PNN coated but non-cholinergic neurons are also indicated. Scalebar: 50 µm.

Topographical pattern of the two motoneuron populations showed similarities in the oculomotor- and trochlear nuclei (Fig. 6A,B), wherein PNN-coated cholinergic neurons were evenly distributed in the volume of the nuclei, whereas non-PNN coated cholinergic neurons were concentrated in the medial part of both nuclei and only one-or-two neurons were scattered in the central or dorso-lateral aspects. In many instances, trochlear motoneurons overlapped with bundles of the MLF. In the abducens nucleus, PNN-bearing motoneurons densely occupy the space ventro-medially to the facial geniculum. The non-PNN bearing cholinergic neurons irregularly occurred in central- or in peripheral localizations (Fig. 6C), incidentally in the proximity of the MLF and facial geniculum or may intermingle with neurons of the reticular formation.

### Two morphological groups of PNNs in the eye movement nuclei of mice

**Figure 7:**
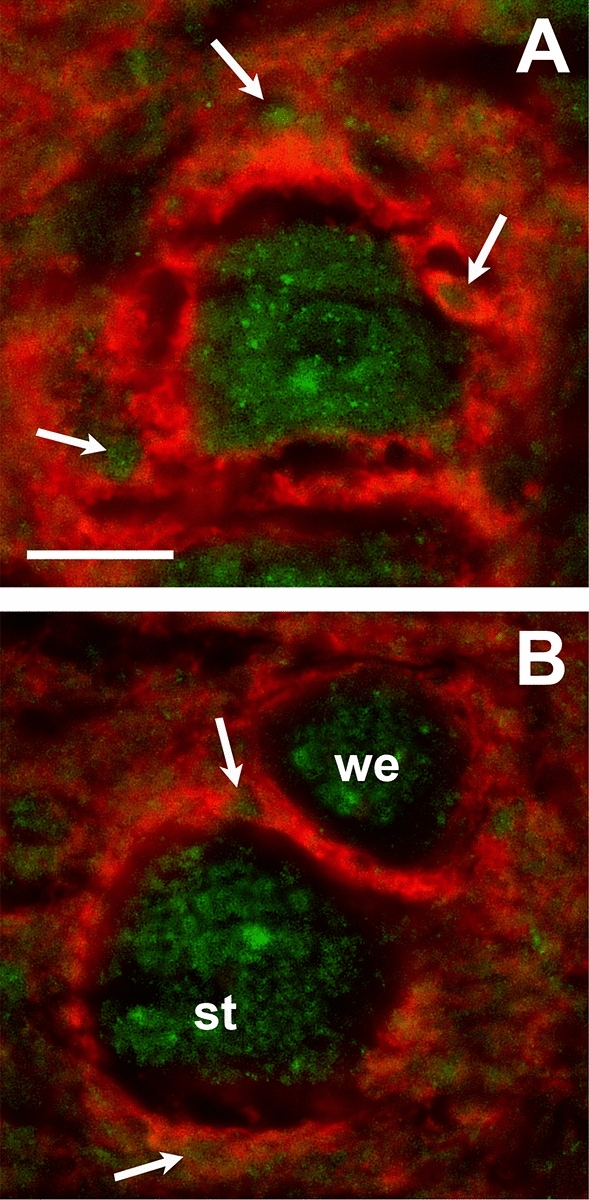
We recognized two morphological types of perineuronal nets in the extraocular motor nuclei of mice, shown with WFA fluorescent labelling. (**A**) Diffuse perineuronal nets spread 6–13 µm towards the neuropil without clear peripheral boundaries. Its spongy structure derives from numerous elliptical tunnel-like forms. Arrows demonstrate ECM tunnels of various diameters, presumably occupied by dendritic- and terminal axon segments. (**B**) Focal perineuronal nets expand max. 1–5 µm distance from the somata and clearly demarcate from the adjacent neuropil. Its compact ECM plates are interrupted by dotted or laminar CSPG accumulations which, in fewer number, also shape tunnel-like ECM sheaths (arrows). st: strongly immunoreactive focal PNN. we: weakly immunoreactive focal PNN. Scalebar: 20 µm.

Both WFA and aggrecan labeling revealed that cholinergic motoneurons were coated by two groups of PNNs, which are distinguished by their morphology. (i) *Diffuse PNNs* (Fig. 7A), also identified around the pyramidal cells of the cortex by Wegner et al.^36^, develop around the minority of eye moving motoneurons, providing 9.2% in oculomotor-, 14.2% in trochlear- and 4.3% in abducent nucleus, of all PNNs covering motoneurons. These PNNs show very high fluorescent intensity and have no demarcated boundary towards the adjacent neuropil. Their generally spongy architecture is constructed of countless circular or ovoid tunnel-like forms (Fig. 7A arrows) in the perisomatic zone. Diameter of tunnels range 1–5 µm and the entire net expands approx. 6–13 µm deep into the neuropil. Some of the ovoid tunnels contained ChAT positive neuronal processes. Diffuse PNNs were evenly scattered centrally and peripherally in the oculomotor nucleus, whereas in the trochlear- and abducens nuclei these were localized mostly in the periphery, with only 1–2 exceptions. (ii) *Focal PNNs* (Fig. 7B) provided the major proportion of perineuronal nets in each of the nuclei, 90.8% in oculomotor-, 85.8% in trochlear- and 95.7% in abducens nucleus. These have either a compact structure, revealing the neuron’s circumference as continuous circles that clearly separates from the near neuropil, or dotted and laminar islets of CSPGs may interrupt those nets, which also form tunnels, of which some are occupied by ChAT positive processes (Fig. 7B arrows). Focal nets envelope the somata, dendrites and axons, and presumably synapses as well. Great variability was recognized among the staining intensities of focal nets and these fluorescent intensities seemed linearly proportionate to their expansions towards the neuropil, reaching max. 1–5 µm thickness from the perikaryon.

### PNNs of cholinergic motoneurons gradually build up in the postnatal period

**Figure 8:**
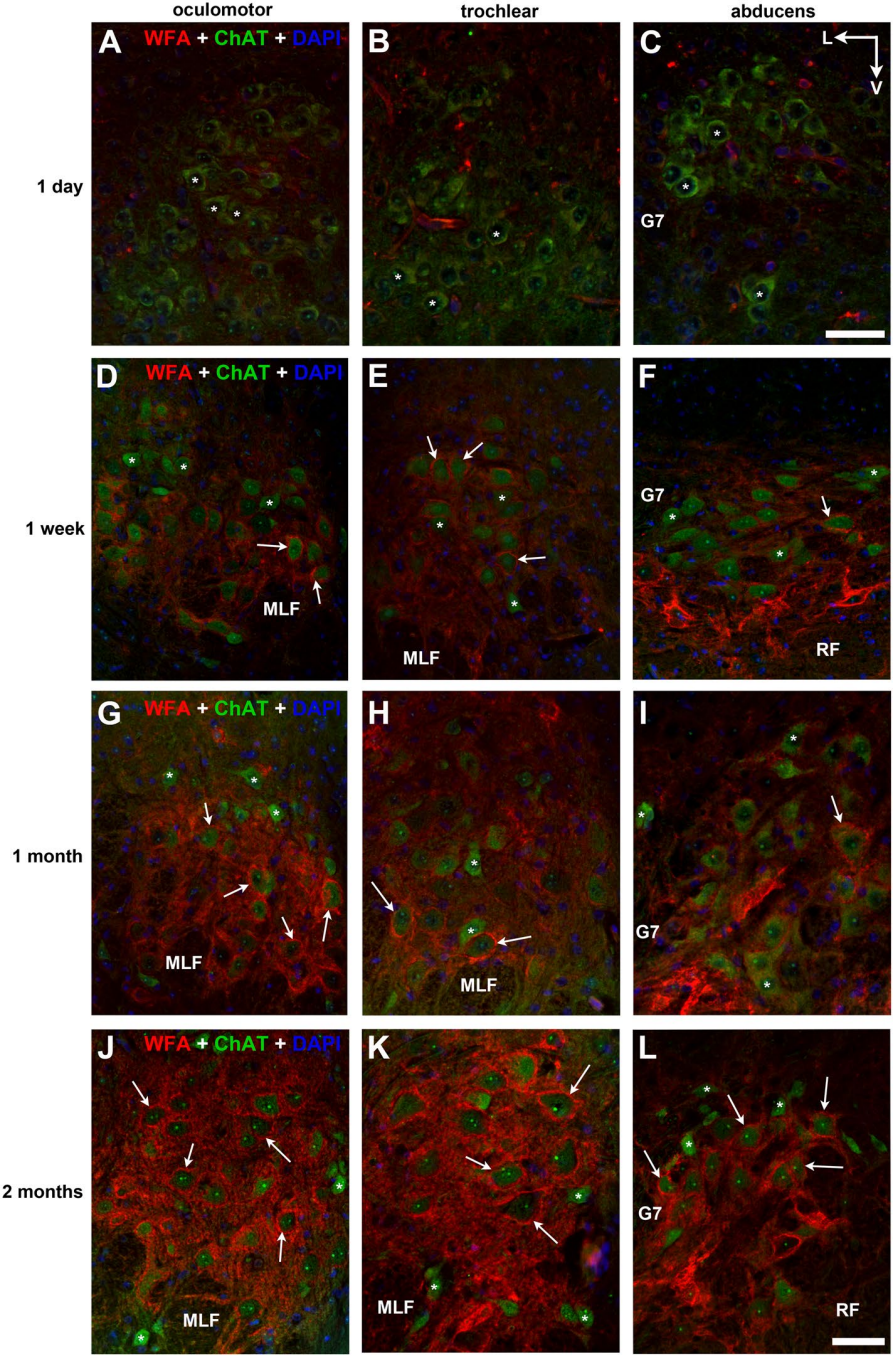
Development of PNNs around cholinergic motoneurons (green) in the oculomotor- (**A**,**D**,**G**,**J**), trochlear- (**B**,**E**,**H**,**K**) and abducens (**C**,**F**,**I**,**L**) nuclei, screened on postnatal 1st day (**A**–**C**), 1st week (**D**–**F**), 1st month (**G**–**I**) and 2nd month (**J**–**L**). PNNs form (labeled with WFA, red) from postnatal day 1, however the proportion of PNN bearing motoneurons (arrows) and histochemical reactivity of PNNs gradually rise towards adulthood. Asterisks: non-PNN bearing motoneurons. G7: facial genu. MLF: medial longitudinal fasciculus. RF: reticular formation. V: ventral. L: lateral. Scalebar: 20 µm.

**Figure 9:**
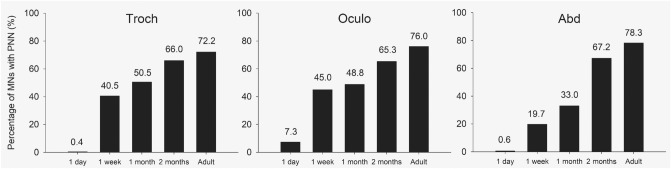
Percentage of PNN bearing cholinergic motoneurons in the extraocular motor nuclei, based on WFA labelling, at different postnatal developmental phases. These proportions of motoneurons with PNNs are significantly different among the developmental phases in all three nuclei (χ^2^(4, n = 1139) = 284.9, p < 0.001; χ^2^(4, n = 1199) = 321.3, p < 0.001 and χ^2^(4, n = 1053) = 346.7 p < 0.001 for the trochlear-, oculomotor- and abducens nuclei, respectively).

With WFA and ChAT co-labeling, we screened the sequence of CSPG accumulation into forming PNNs around cholinergic motoneurons. On the 1st postnatal day, we have only recognized very few pale stained perineuronal nets in the oculomotor nucleus (Fig. 8A); but the neuropil was devoid of proteoglycan accumulation. We also noticed the small diameter of cholinergic somas at the time of birth (8–15 µm), which dramatically enlarged after 1 week. At 1 week age, the proportion of PNN bearing motoneurons raised to 45% in the oculomotor-, 40.5% in trochlear- and 19.7% in abducens nucleus (Figs. 8D–F and 9); also, the fluorescens of neuropils in each nucleus has strongly raised during the first PN week. At 1 month age, the ratio of PNN-bearing vs. non-PNN-bearing motoneurons turned nearly equal in the oculomotor- (48.8%) and trochlear nuclei (50.5%), whereas it was only 33% in the abducens nucleus (Figs. 8G–I and 9). At 2 months age, PNN bearing motoneurons made-up 65.3% in oculomotor-, 66% in trochlear- and 67.2% in the abducens nucleus of all ChAT positive cells (Figs. 8J–L and 9). The chi-square test showed that proportions of motoneurons with PNNs are significantly different among the developmental phases in all three nuclei (oculomotor: χ^2^(4, n = 1199) = 321.3, p < 0.001; trochlear: χ^2^(4, n = 1139) = 284.9, p < 0.001; abducens: χ^2^(4, n = 1053) = 346.7 p < 0.001). Due to the several-fold lower histochemical reactivity in early developmental stages—compared to adult animals—, identical microscope and software adjustments could not be applied during microphotography.

A scattered population of non-cholinergic PNNs were also recognized in the abducens nucleus already on postnatal week 1, and in the oculomotor- and trochlear nuclei as well at 1 month age. The early development of these neurons is presumably important in internuclear network establishment between brainstem centers. These interneurons remain in adulthood, whose PNNs’ immunoreactivity was highly beyond that of SIF motoneurons (Figs. 1A,B, 2A,B, 3A,B).

We were also curious about the progression of focal and diffuse nets in a developmental perspective. On PN day 1 and week 1 we recognized only focal PNNs around cholinergic motoneurons. Diffuse type PNNs were firstly seen at 1 month age in the trochlear nucleus, providing approx. 2.7% of PNNs, which ratio increased to 7% by the 2nd month. In the oculomotor- and abducens nuclei diffuse nets developed only during the 2nd month, providing 8.3% and 3% of cholinergic PNNs at the time of sampling, respectively.

## Discussion

The present neuro-morphology- and morphometry-based study focused on the distribution of lecticans in PNNs and neuropil of the oculomotor-, trochlear- and abducens nuclei in mice and attempted to find possible associations with earlier neuroanatomical works classifying extraocular motoneurons which enable the utmost precise control of visuomotor kinetics.

The anti-choline acetyltransferase immunolabeling in each section was combined with WFA labeling and antibodies against lecticans specifically, which allowed us to recognize three neuron types in the extraocular motor nuclei, the SIF- and MIF motoneurons, and presumably internuclear neurons too, the latter represented by the scattered non-cholinergic PNNs. Specifically, taking the matrix into analysis, we found evidence for the presence of both diffuse ECM throughout the neuropil of the nuclei and condensed matrix forms were too present, thus the PNNs, axonal coats and perisynaptic matrix accumulations. Outside our enquiry, we also noticed the nodal ECM in the facial genu and MLF, which are micro-accumulations of CSPGs, indirectly contributing to saltatory propagation of action potentials.

Based on our findings we concluded that SIF- and MIF motoneurons do not segregate sharply in mice, which seems conservative in afoveolates, as is has also been recognized in rats^20^. Moreover, our findings suggest that the pattern of SIF and MIF segregation is specific to each nucleus. The majority of extraocular motoneurons are the PNN coated A- and B-motoneurons, which have been identified as SIF motoneurons by their histochemical properties in monkey^23^, rat^20^, and in mouse^22^. In each of the extraocular motor nuclei, SIFs of individual muscles separate into clusters, which groups have not been re-mapped in the present study, nevertheless the spatial distribution of MIF motoneurons was found accordingly. ***nuIII.*** We found the population of ChAT positive motoneurons without PNN coverage loosely scattered at the medial margin of the oculomotor nucleus, as also seen in rats by Eberhorn et al.^20^. Considering the histochemical properties and localization described by other studies, we suggest these motoneurons as C- and S- groups of MIFs. Compared with frontal eyed animals, like cat and monkey, this arrangement appeared very much conservative across species (^23^(monkey)^37^ (cat)). However, in mice, MIFs and SIF motoneurons do not isolate as strictly, as they do in cats and monkeys, in fact considerable numbers of ChAT positive- but PNN lacking cells scatter within the oculomotor nucleus proper. Comparing the ratio of putative SIFs to MIFs (4.2:1) in mice, we calculated a 24% of MIF motoneurons among all cholinergic cells, which number is similar to those identified in tracer experiments in the monkey and rat. ***nuIV***. In the trochlear nucleus we could not identify the strong dorsal cap of MIF motoneurons, as seen in monkeys^23^, in fact, the majority of PNN lacking motoneurons were seen at the medial boundary of the nucleus and, in small numbers, mixed with PNN coated neurons in the center of the nucleus. The ratio of SIFs to MIFs was 28% (3.6:1), which is a slightly higher value in comparison to monkeys and nearly double than in rats. The functional background of this recognition is, most likely, the particularly developed oblique muscles in the mouse^22^, which rodent specialty is in the favor of counter torsional movements of the eyes during head rolling in these laterally eyed species^38,39^. ***nuVI***. In the rat, majority of MIF motoneurons are grouped into a strong cluster close to the medial and dorsal border of the abducens nucleus^20^. Opposite to that finding, in the mouse we could not identify this pattern of MIFs. Many ChAT positive neurons without PNN coverage lied outside the medial and dorsal borders of the nucleus. We have also seen similar motoneurons ventrally and centrally, thus we consider their distribution rather incidental than strictly clustered. The ratio of SIFs to MIFs was 22% (4.6:1), which value closely matches with those seen in monkeys and rats.

Considering the diameters of ChAT positive extraocular motoneurons of mice, our data supports the report by Bohlen et al.^22^ who did not find any significant difference between the sizes of SIFs and MIFs somata either. However, this contrasts with what was seen in primates^23,24^ or rats^20^.

A major finding of our work is that the mostly dominating molecular constituent of PNNs coating extraocular motoneurons, is aggrecan. The WFA lectin binds to N-acetyl galactosamine substituents of CSPGs, which are present in highest numbers on the core protein of the aggrecan^40^. The strong PNNs recognized with aggrecan immunoreaction and absence of PNNs in the brevican, neurocan and versican co-labelled sections support our finding.

In several regions of the CNS, such as in the vestibular nuclei^2^, red nucleus^41^, or in the olfactory brain^42^ brevican, neurocan and versican were recognized as major constituents of PNNs. Contrarily, in the PNNs of extraocular motoneurons none of those lecticans accumulate. Moreover, we found cytoplasmic brevican immuno-signal consistently in each nucleus providing a yellowish discoloration of somata. Such co-labeling was not unexpected, as the cytoplasmic presence of brevican was found previously in the vestibular nuclei of the rat^2,43^. The micro-accumulations of versican were not unexpected either; we suggest that those represent the perisynaptic and periaxonal matrix.

Optical density measurements of PNNs showed the highest values in the trochlear nucleus with WFA labeling and in the abducens nucleus with aggrecan labelling, and the lowest averages for both reactions were calculated in the oculomotor nucleus. At present, we cannot provide an irrefutable description on the local function of such strong PNNs. Speculations on possible biological significance of strong PNNs in nuIV may lead us to note that superior oblique muscle in mice is highly developed due to compensatory eye elevation during head tilting, which motion is not analogously present in frontal eyed oculokinetics^22^. The relatively weak optical densities in PNNs of nuIII could be explained by the wider range of afferentations terminating on dendrites of motoneurons and the simultaneous presence of SIF-, MIF- or internuclear neurons, and other physiological groups; the latter yet poorly described^23^. The intensive aggrecan immunoreaction exceeding WFA fluorescence in the abducens nucleus was unforseen. Various explanations may support this finding: (i) physiological properties that complement rapid tilting movements of the head in the coordination of saccadic eye movements during seeking; (ii) the more compact structure of the nucleus; (iii) or less N-acetylgalactosamin substituents accessible for WFA lectin.

Mice are born with an underdeveloped oculokinetic system. Brainstem motoneurons are born relatively early though (E10-13), their integration into the cortico-nuclear tract only begins on the first PN week^44^. The small size of motoneurons and very compact arrangement of all eye movement nuclei in newborn animal prove that their dendritic arbor and hodology is yet to form. The optic tract myelinates from PN day 8 establishing eyesight before eyelids open on PN day 12. Integration of the oculomotor network happens meanwhile (Figs. 8 and 9), which process is supported by the acceleration of synaptogenesis and pruning between PN days 3–20^44,45^. CSPG accumulation in extracellular spaces into PNNs marks the timepoint of synaptic maturity and structural stabilization^46,47^.

We identified two morphological types of PNNs around extraocular motoneurons ubiquitous in the three extraocular motor nuclei of mice. We recognized 90% of PNNs as focal nets, and only 10% of PNNs had diffuse boundaries. We also noticed that immunoreactivity of diffuse nets was strongly beyond the optical densities of focal nets. Both in the focal and diffuse nets we could notice holes or rather tunnels, some of which contained ChAT immuno-positive processes, whereas other tunnels remained unstained. These latter ones are presumably the axonal coats of afferents from the ipsi- and contralateral MLF, pontine paramedian reticular formation, prepositus hypoglossi nucleus, supraoculomotor regions^48^, internuclear inputs, or GABAergic axon terminals from the superior vestibular nucleus^49^.

Diffuse PNNs were firstly termed by Wegner et al.^36^ as a unique entity around a subtype of pyramidal cells in the cerebral cortex of rats, associated with partially different cytochemical, physiological and structural properties^50,51^ to whom diffuse PNNs provide a special operational environment^52^. With the presently used methods however, we can merely assume similar functions/characteristics of the diffuse PNNs associated with this system.

The present morphology-based study concluded that PNNs of extraocular motoneurons are dominantly composed of aggrecan, while accumulation of other lectican molecules is limited to the neuropil, where they presumably accumulate as axonal coats, peridendritic and perisynaptic matrix. Optical density values of WFA and aggrecan stained PNNs showed the highest intensities in the trochlear- and abducens nuclei, respectively. The functional explanation of this finding remains speculative, yet we suggest the highly developed oblique muscles and specific eye movements, seen in afoveolates like rats and mice, to be in the background. We recognized SIF- and MIF motoneurons by applying histochemical descriptions of earlier works. The distribution of MIFs showed similarities with other lateral eyed animals like rats, and to some extent with foveolates as well. Nevertheless, mice do exhibit a specific distribution of both SIFs and MIFs, especially in the trochlear nucleus, where, in comparison to rats or primates, we found the considerably higher number of ChAT positive neurons without PNN coverage. We have also classified PNNs into focal and diffuse morphological groups, although their possibly differing functional properties in related neuronal networks must be investigated by further studies. For now, we propose focal and diffuse PNNs to reflect on different repertoire of voluntary eye movements in functionally afoveolate (rats, mice) and foveolate animals (monkey)^20,53–55^.

## Methods

### Animals and tissue harvesting

Observations were performed on six adult female (12–14-week-old) NMRI mice, weighing from 25 to 30 g, and on young animals at postnatal 1st day, 1st week, 1st month and 2nd month, using two animals to each phase. Animals had unlimited access to food and water, and constant temperature (23 °C) and humidity were provided in 12 h light-and-dark cycles. Experimental procedures were approved by and complied with guidelines of the ‘University of Debrecen–Committee of Animal Welfare’ (license number: 6/2017/DEMAB) and the ‘Scientific Ethics Committee of Animal Experimentation’ (number: HB/06/ÉLB/2270-10/2017; June 6, 2017). Housing and treatment of animals strictly met regulations of the European Union [European Communities Council Directive of 24 November 1986 (86/609/EEC)]. Furthermore, experiments were performed in accordance with the ARRIVE guidelines. Every opportunity was taken to reduce the number and suffering of animals.

Narcotized mice were terminally anesthetized with the intraperitoneally introduced mixture of 10% ketamine (100 mg/kg; CP Pharma Handels GmbH, Burgdorf, Germany) and 2% xylazine (10 mg/kg; Produlab Pharma BV, Raamsdonksveer, Netherlands). Subsequently to transcardial perfusion with physiological saline, brainstems were immediately removed and fixed by immersion in Sainte Marie’s fixative (1% glacial acetic acid in 99% abs. ethanol) at 4 °C, overnight. Brainstems were embedded into paraffin and transverse sections were made at the thickness of 8 µm.

### Histochemistry and immunohistochemistry

We applied double fluorescent immunolabeling on each section to match acetyl-cholinergic motoneurons with the above listed forms of the ECM. Deparaffinated sections were rehydrated and washed in phosphate buffered saline pH 7.4 (PBS) and immersed into 3% H_2_O_2_ for 10 min at room temperature (RT). Antigenic sequences of aggrecan, brevican and vesican were exposed by digesting sections with chondroitinase ABC (1:100; 0.02 U/mL; Sigma-Aldrich, St. Louis, MO, USA) diluted in specific Tris sodium-acetate buffer, pH 8.0 for 1 h at 37 °C. Tissue samples were blocked for 1 h at RT in 2% bovine serum albumin (BSA) (*Wisteria floribunda* agglutinin; WFA), or 10% normal goat serum (NGS) + 1% BSA (aggrecan); or 10% normal donkey serum (NDS) + 1% BSA (neurocan, brevican, choline acetyltransferase); or 20% NGS + 2% BSA (versican).

Cholinergic motoneurons in each section were labeled with anti-choline acetyltransferase primary antibody (ChAT; goat, Merck-Millipore, Temecula, CA, USA; RRID:AB_2079751) diluted in PBS with 3% NDS + 1% BSA at 4 °C, overnight. Reaction was visualized with donkey anti-goat IgG AlexaFluor 488 (Invitrogen; Eugene, OR, USA; RRID:AB_2534102) in PBS at RT for 1 h. ChAT labeling was coupled with one of the following histochemical or immunohistochemical reactions: N-acetylgalactosamin substituents of chondroitin sulfate proteoglycans were labeled with biotinylated WFA lectin (Sigma-Aldrich, St. Louis, MO, USA ; RRID:AB_2620171), dissolved in PBS containing 1% BSA for one overnight at 4 °C, visualized with Streptavidin AlexaFluor 555 (Invitrogen; RRID:AB_2571525) secondary reagent, incubated in PBS for 1 h at RT; or anti-aggrecan (rabbit; Merck-Millipore; RRID:AB_90460) diluted in PBS containing 3% NGS + 1% BSA at 4 °C overnight, then visualized with goat anti-rabbit AlexaFluor 555 (Invitrogen; RRID:AB_2535850) diluted in PBS at RT for 1 h; or anti-brevican (sheep; R&D Systems, Minneapolis, MN, USA; RRID:AB_2274793) in PBS containing 3% NDS + 1% BSA at 4 °C, overnight, then visualized with donkey anti-sheep AlexaFluor 555 (Invitrogen; RRID:AB_2535857) diluted in PBS at RT for 1 h; or anti-neurocan (sheep; R&D Systems; RRID:AB_2149717) diluted in PBS containing 3% NDS + 1% BSA at 4 °C, overnight, then visualized with donkey anti-sheep AlexaFluor 555 (Invitrogen; RRID:AB_2535857) diluted in PBS at RT for 1 h; or anti-versican (rabbit; Biorbyt, Cambridge, UK) diluted in PBS containing 3% NGS + 1% BSA at 4 °C for three overnights, then visualized with biotinylated goat anti-rabbit IgG (Vector Laboratories, Burlingame, CA, USA; RRID:AB_2313606) for 1 h at RT and StreptAvidin AlexaFluor 555 (Invitrogen; RRID:AB_2571525) at RT for 1 h, both diluted in PBS (Tables 1 and 2). Each section was cover slipped with Prolong^®^ Diamond Antifade mounting medium (Invitrogen).

**Table 1:** Lectin and primary antibodies used.

	bWFA^a^	Anti-aggrecan	Anti-brevican	Anti-neurocan	Anti-versican	Anti-choline-acetyltransferase
Supplier, cat. no.	Sigma-Aldrich; L1516	Merck Millipore; AB1031	R&D Systems; AF4009	R&D Systems; AF5800	Biorbyt; orb259630	Merck Millipore; AB144P
Species of origin, type	Lectin isolated from *Wisteria floribunda;* biotinylated	Rabbit; polyclonal, IgG	Sheep; polyclonal, IgG	Sheep; polyclonal, IgG	Rabbit; polyclonal, IgG	Goat; polyclonal, IgG
Immunogen	-	GST fusion protein containing amino acids 1177–1326 of mouse aggrecan	Mouse myeloma cell line NS0 derived recombinant human brevican Asp23-Pro911	Chinese hamster ovary cell line CHO derived recombinant mouse Neurocan Asp23-Asp637	A synthetic peptide corresponding to a sequence at the N-terminus of human Versican	Human placental enzyme
Dilution	1:500	1:500	1:50	1:300	1:200	1:100

^a^Biotinylated *Wisteria floribunda* agglutinin.

**Table 2:** Fluorescent secondary antisera.

Antisera	Species of origin	Supplier, cat. No.	Dilution
Anti-goat IgG AlexaFluor 488 (choline acetyltransferase)	Donkey	Invitrogen, A11055	1:1000
		RRID:AB_2534102	
Anti-rabbit IgG AlexaFluor 555 (aggrecan)	Goat	Invitrogen, A21429	1:1000
		RRID:AB_2535850	
Anti-sheep IgG AlexaFluor 555 (brevican, neurocan)	Donkey	Invitrogen, A21436	1:1000
		RRID:AB_2535857	
Biotinylated anti-rabbit IgG (versican)	Goat	Vector Laboratories, BA-1000	1:200
		RRID:AB_2313606	
Streptavidin AlexaFluor 555 (bWFA, versican)	–	Invitrogen, S32355	1:1000
		RRID:AB_2571525	

Fluorescent photographs were captured using Olympus CX31 epifluorescent microscope equipped with Olympus DP74 camera (both manufactured by Olympus, Tokyo, Japan). Comparability of fluorescent signals has been ensured by standardized hardware and software adjustments of the microscope setup, while recording images of the same reactions. Final diagrams were edited with Adobe Photoshop CS4 (Adobe Systems Inc., San Jose, CA, USA).

### Analysis of fluorescent staining intensity

For quantification of immunofluorescence, optical densitometry of PNNs was made by a third experimenter with ImageJ v1.46 software (National Institutes of Health, Bethesda, MD, USA). Staining intensity of PNNs was quantified as performed recently by Magyar et al. (2022). Briefly, in single channel 555-nm-excited monochrome frames the PNNs were drawn out manually, then the average intensity of fluorescent pixels was calculated by the software on 8-bit scale (0–255 range). We exclusively quantified the WFA (309 PNNs in total; 76–122 per nucleus) and aggrecan (503 PNNs in total; 97–235 per nucleus) labeled PNNs of ChAT positive neurons, since reactions against other lecticans did not outline any perineuronal nets, or only dotted perineuronal accumulations were recognizable. Background intensity was subtracted from PNN values by the software, sampled in an unstained area of the reticular formation.

Based on their morphology, perineuronal nets of cholinergic motoneurons were classified into two distinct morphological groups, considering to the following criteria: demarcation from neuropil; thickness; compact or spongy architecture; intensity of immunofluorescence.

### Statistical analysis

Optical densities were statistically analyzed to compare PNNs of the three eye movement nuclei. Necessary sample size was calculated by the WebPower^21^ software prior to the start of the study. The input parameters of this power analysis for the future One-Way Analysis of Variance (ANOVA) tests were: number of groups = 3, effect size = 0.2, significance level = 0.05, required power = 0.8. Power analysis yielded 244 as the required minimum sample size. Real sample sizes (number of PNNs analyzed) in the study were above this minimum requirement, 309 and 503 for the WFA and aggrecan reactions respectively. Statistical analysis on collected data was performed by the SigmaPlot for Windows v14.0 (Systat Software, Inc., San Jose, CA, USA) software package. Normality of frequency distributions of optical densities and equality of variances were tested by Shapiro–Wilk and Brown-Forsythe tests respectively. Optical densities of WFA and aggrecan immunohistochemical signals of PNNs among different nuclei in adult animals were compared by the non-parametric Kruskal–Wallis One Way Analysis of Variance (ANOVA) on Ranks test. Alpha level for these tests was 0.05. Pairwise comparisons of optical reaction intensities of PNNs in different nuclei were performed by the Dunn’s post hoc test. Differences were considered statistically significant at the level of p < 0.05.

Optical densities were normalized by the mean optical density of PNNs in the trochlear nucleus (100%) and were graphed by the SigmaPlot for Windows v14.0 (Systat Software, Inc., San Jose, CA, USA) software. In box plots, the number of asterisks mark different p levels in pairwise comparisons, *** p < 0.001, ** 0.001 < p < 0.01 and * 0.01 < p < 0.05. Numerical values are means ± standard errors.

Ratios of PNN-bearing- and non-bearing cholinergic motoneurons in different nuclei were compared by the χ^2^-test.

### Ratio and topography of PNN bearing and non-bearing cholinergic motoneurons

On double stained sections the exact numbers of PNN bearing- and lacking cholinergic motoneurons were counted in each nucleus (175–271 neurons per nucleus) to compare their ratios among the nuclei. Only those somata were taken into count which showed clear signals of both WFA and anti-ChAT reactions, presenting the diameter of 10 μm or above, to exclude uncertain cell fractions.

Earlier attempts have been made to determine if SIF motoneurons segregate from MIFs in rodents^22^, as seen clearly in primates^20^, although evidence is still lacking to prove that. Graphical reconstructions have been made with ImageJ software to illustrate topography of the two neuronal populations within each nucleus.

## Data Availability

The data that support the findings of this study are available from the corresponding author, upon reasonable request.
